# Populations of *Heterodera schachtii* Differ in Susceptibility to Rhizosphere Bacteria Structured by Plant Age

**DOI:** 10.3390/microorganisms13020289

**Published:** 2025-01-28

**Authors:** Rasha Haj Nuaima, Eva Tanneau, Holger Heuer

**Affiliations:** 1 Institute for Epidemiology and Pathogen Diagnostics, Julius Kühn Institute, 38104 Brunswick, Germany; 2 Max Planck Institute for Evolutionary Biology, 24306 Plön, Germany; etanneau@bot.uni-kiel.de

**Keywords:** *Heterodera schachtii*, microbes, plant’s age, populations, resistant oilseed radish, rhizosphere

## Abstract

Rhizosphere microbes, particularly bacteria, are essential for controlling plant-parasitic nematodes (PPNs) through various mechanisms. However, the plant’s age and the genetic composition of nematode populations can significantly influence the inhibitory effectiveness of these microbes against the beet cyst nematode *Heterodera schachtii*. In this study, rhizosphere microbes were isolated from 39-day-old and 69-day-old resistant oilseed radish plants to evaluate their impact on the penetration of the second-stage juveniles (J2s) originating from four genetically distinct *H. schachtii* populations. The suppression of J2s penetration by the attached microbes varied across the nematode populations, which displayed differing levels of aggressiveness toward the resistant oilseed radish. Furthermore, differences in the alpha and beta diversity of rhizosphere bacteria were observed between the 39-day-old and 69-day-old plants, leading to variations in the bacterial attachment among the four nematode populations. In summary, the effectiveness of resistant catch crops against *H. schachtii* is influenced by the pathogenicity of the nematode populations and their interactions with the rhizosphere microbial community shaped by the plant’s age.

## 1. Introduction

Rhizosphere-associated microorganisms, including bacteria, fungi, and other microbes are pivotal in suppressing plant-parasitic nematodes (PPNs) through direct and indirect mechanisms [1,2,3,4].

These microorganisms inhabit the soil surrounding plant roots, where they interact with nematodes, promoting plant health and playing a key role in controlling plant-parasitic nematodes (PPNs) [3,4,5,6,7,8,9].

Certain rhizosphere bacteria, such as *Pseudomonas fluorescens* (Proteobacteria), produce secondary metabolites like 2,4-diacetylphloroglucinol (DAPG), hydrogen cyanide (HCN), and pyoluteorin, which have toxic effects on nematodes. These compounds impair nematode mobility and their ability to penetrate plant roots, thus reducing nematode infestations [10].

*Bacillus thuringiensis* (Firmicutes) also secretes crystal proteins (Cry), which are harmful to invertebrates, including nematodes. After the ingestion, these proteins become activated in the nematode’s gut, leading to cell lysis and nematode mortality [11,12].

*Streptomyces* species (Actinobacteria) produce a variety of antibiotics, such as avermectins and milbemycins, which possess nematicidal properties. These compounds disrupt the nervous system and metabolic functions of nematodes, further contributing to their suppression [13].

In addition to the toxic effects of specific metabolites, beneficial microbes can outcompete PPNs for space and resources in the rhizosphere. For instance, *Bacillus subtilis* can aggressively colonize the root surface and rhizosphere, forming biofilms and depleting available nutrients, thus limiting the resources available for nematode colonization [14].

Fungal species like *Pochonia chlamydosporia* can parasitize the eggs and juveniles of beet cyst nematodes (*H. schachtii*), significantly reducing their population density in the soil [15,16].

While rhizosphere microbes are widely acknowledged in suppressing PPNs, their effectiveness can vary significantly depending on plant factors such as age and growth stage. The developmental stage of a plant significantly impacts the structure, diversity, and functionality of the microbial community in its rhizosphere. Younger plants typically release root exudates that differ in composition from those of mature plants, shaping distinct microbial populations [17].

Consequently, the composition and activity of rhizosphere microbes dynamically adjust throughout the plant’s growth stages, reflecting changes in root exudation patterns [18,19,20,21].

The efficacy of microbial control also varies with the genetic structure of the nematode population. Studies have shown that soil microbes exhibit varying suppressive effects on root lesion nematodes (*Pratylenchus neglectus*) with different genetic structures [22].

In addition, bacterial attachment by *Pasturia penetrans* has been shown to vary among *Meloidogyne* populations [23]. The research on *M. javanica*, *M. incognita*, and *M. hapla* revealed that attachment specificity occurs at the subspecies level, with significant differences in the number of attached endospores observed among populations of the same nematode species. A substantial genetic variability has also been identified within and among geographical populations of the beet cyst nematode, *H. schachtii* [24,25,26]. However, the impact of nematode population genetic structure on the effectiveness of microbial agents in suppressing this nematode remains unstudied. Advancing knowledge in this area could provide valuable insights for managing *H. schachtii*, which is responsible for significant crop losses across a broad host range, including sugar beet and species from 23 plant families [27]. To this point, understanding how microbial effects change in response to plant age and the genetic structure of nematode populations is essential for improving strategies to manage plant-parasitic nematodes.

In this study, we aimed to carry out the following:

1. Investigate the effect of rhizosphere microbes from resistant oilseed radish plants at 39 and 69 days of age on the penetration of J2s from four genetically distinct populations of *H. schachtii* using Pluronic F127 gel assays.
2. Analyze the composition of rhizosphere bacterial communities at these two plant ages using Illumina sequencing.

## 2. Material and Methods

### 2.1. Nematode Culture and J2-Surface Sterilization

Populations of the beet cyst nematode *H. schachtii* were collected from four locations in Germany and Ireland. The cysts were extracted from soil samples using a magnesium sulfate (MgSO_4_) solution [27]. Species identification was performed by PCR amplification, followed by sequencing of the cytochrome c oxidase subunit 1 (*COI*) gene. Cysts from each population were reared on a susceptible cabbage (*Brassica oleracea* L., cv. *storema*) for two generations and stored in loess substrates.

Based on published [25] and unpublished data regarding the gene patterns of *vap1* for *H. schachtii*, four nematode populations with differing *vap1* structures were selected. These populations originated from two regions in Germany, North Rhine-Westphalia (Kerpen-Buir, Elsdorf) and Bavaria (Acholshausen), and one from Ireland.

To extract cysts, 100 g of loess substrate was washed with tap water in a bucket fitted with a 250 µm sieve. The cysts collected on the sieve were placed on filter paper for the hatching process.

For each population, 1500 cysts were placed on a fleece filter in a hatching solution of ZnCl_2_ (408 mg/L) [28]. The juveniles that migrated downward through the filter were collected and subjected to sterilization.

The surface sterilization of J2s was performed following a modified version of the protocol described by Hay (1994) [29]. Briefly, J2s were immersed in a 0.005% Chlorhexidine solution for 15 min, rinsed with sterilized water on a 5 µm sieve, and then placed in an antibiotic solution containing Penicillin-G (0.1% *w*/*v*) and Streptomycin sulfate BP (0.1% *w*/*v*) for 16 h. Afterward, they were washed threefold with 15 mL of sterilized water on a 5 µm sieve and stored at 4 °C. The Chlorhexidine and antibiotic incubations were carried out in 10 mL glass tubes rotating at moderate speed using a Tube Rotator and Mixer (OCS Tech, Neuching, Germany).

### 2.2. The Bacterial Attachment Test

The resistant oilseed radish (*Raphanus sativus* L., var. *colonel*) was planted in pots containing field soil under greenhouse conditions (20/16 °C with a 16/8 h day/night cycle). Plants aged 39 and 69 days were harvested for isolating the rhizosphere microbes. Root samples were shaken threefold in 20 mL of sterilized water using a Stomacher (Fisher Scientific, Schwerte, Germany) at 300 rpm for 1 min. After centrifugation at 500× *g* for 5 min at 4 °C, the supernatant was passed through a 5 µm sieve to remove any remaining nematodes. The bacterial solution was adjusted to an OD600 of 0.2 for the attachment test.

J2s were surface-sterilized following the procedure described above. To eliminate microbial contaminants from the nematode cuticle, J2s were treated with a 0.005% Chlorhexidine solution for 15 min, rinsed three times with sterilized water using a 5 µm sieve, and incubated at room temperature for 24 h to activate the attachment potential of the cuticle. Approximately 3500 J2s from each population were incubated overnight in 3 mL of microbial suspension at 28 °C on a shaker set at 150 rpm. As a control, an additional batch of 3500 surface-sterilized J2s was maintained in 3.5 mL of sterilized water under the same conditions.

J2s were recovered on sterile 5 µm sieves and washed threefold with 15 mL of sterile water, then transferred to 15 mL tubes containing 3.5 mL of sterile water. For the attachment test, 12 replicates of 15 J2s each, incubated in the microbial rhizosphere of 39-day-old oilseed radish, were plated on R2A media (Merck, Darmstadt, Germany) and incubated at 28 °C for 2 days. The colony-forming units (CFU) formed by live bacteria attached to the nematode cuticle were counted for each treatment. Surface-sterilized J2s without baiting served as a control treatment.

### 2.3. Bioassay to Evaluate the Penetration of H. schachtii Populations into Oilseed Radish Roots with and Without the Microbial Attachment

Seeds of oilseed radish (*Raphanus sativus* L., var. *colonel*) were surface-sterilized by soaking in 2% sodium hypochlorite solution for five minutes, followed by three washes with sterilized water. For germination, the sterilized seeds were placed in a Petri dish containing one-third-strength PGA medium (X931.1 Carl Roth) and incubated at room temperature for six days.

The oilseed radish seedlings were transferred to divided Petri dishes (100 mm × 15 mm, Roth, Graz, Austria) containing 15 mL of 23% Pluronic F127 gel (Sigma-Aldrich, St. Louis, MO, USA). The gel was prepared according to the protocol described by Williamson and Čepulytė (2017) [30] to assess nematode penetration into the roots of oilseed radish.

For each population, 3000 J2s were incubated overnight at 28 °C in a glass tube containing either 3 mL of rhizosphere microbial suspension (OD600 = 0.2) or 3 mL of sterilized water.

J2s exposed to the microbial suspension were washed threefold with sterilized water using a 5 µm sieve and transferred into a new glass tube.

Within each treatment and for each population, 500 J2s with six replicates, either baited in sterilized water or in a microbial suspension, were added to the oilseed radish seedlings. The seedlings were incubated at 22 °C and 60% relative humidity. Seven days after nematode inoculation, the roots of the oilseed radish seedlings were harvested and stained with acid fuchsin solution, as described by Nuaima et al. (2019) [31]. This bioassay was conducted using rhizosphere microbes isolated from either 39-day-old or 69-day-old oilseed radish plants (var. Colonel).

### 2.4. Amplifying *16S rRNA* Gene of Attached Bacteria for Sequence Determination

The microbial DNA extracted from the rhizosphere of 39- and 69-day-old oilseed radish, as well as from the microbe-attached J2s of the four studied populations, was isolated using the Fast Prep FP120 bead-beating system (MP Biomedicals, Santa Ana, CA, USA) for 30 s at 6.0 m/s, followed by the Fast DNA Spin Kit for Soil (MP Biomedicals). The Phyla of the rhizosphere bacteria and those attached to J2 were identified by sequencing the *16S rRNA* gene using an Illumina MiSeq sequencer (NGS, MiSeq, NOVOGENE, London, UK). A total of 10,000 sequences per sample were obtained, with four replicates per condition. The sequences were processed and assigned to operational taxonomic units (OTUs) for further analysis.

### 2.5. Data Analysis

The analysis of CFU data for attached bacteria and the number of J2s that penetrated oilseed radish roots was performed using the R statistical 4.0.4 software packages. The Wilcoxon test was performed following a significant Kruskal–Wallis test, whereas the *t*-test was used to compare group pairs with normally distributed data. Illumina sequencing data were processed using a pipeline developed by NOVOGENE (London, UK)to demonstrate the relative abundance, alpha- and beta-diversity of the analyzed bacterial communities.

## 3. Results

### 3.1. Attachment of Rhizosphere Bacteria from 39-Day-Old Plants to the J2 Cuticle of H. schachtii Populations

**Figure 1:**
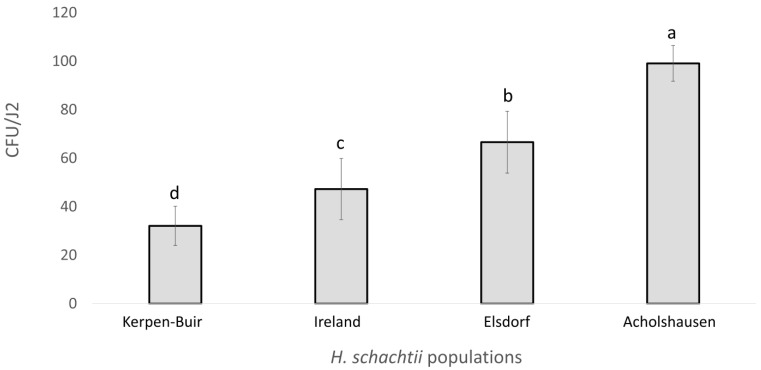
The bacterial attachment to J2s originated from four populations of *H. schachtii* (Kerpen-Buir, Elsdorf, and Acholshausen from Germany, and one from Ireland). The number of colony-forming units (CFU) formed by bacterial cells attached to J2 was determined after incubation at 28 °C overnight in the rhizosphere suspension of 39-day-old resistant oilseed radish “Colonel”. Different letters indicate significant differences in the counts of CFU/J2 among populations (Wilcoxon test, *t*-test, *p* < 0.05, *n* = 12).

The assay revealed significant differences in the number of bacterial cells isolated from the rhizosphere of resistant oilseed radish “Colonel” and attached to the J2 cuticle among the four geographically distinct populations of *H. schachtii*. The Acholshausen population exhibited the highest average number of CFUs per individual, while the Kerpen-Buir population showed the lowest (Figure 1).

The variability in CFU counts among individuals was greater in the Kerpen-Buir and Ireland populations compared to the Elsdorf and Acholshausen populations. Specifically, the CFU count range was 7–91 in the Elsdorf population and 69–136 in the Acholshausen population. In contrast, the range was narrower in the Kerpen-Buir population (5–19) and the Ireland population (5–37).

### 3.2. The Penetration of H. schachtii Populations into Oilseed Radish Roots, with and Without Attachment of Rhizosphere Microbes from 39-Day-Old Plants

**Figure 2:**
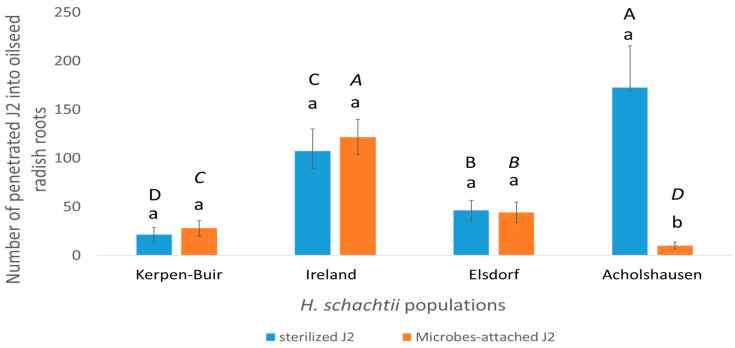
The effect of the rhizosphere microbes isolated from 39-day-old plants of resistant oilseed radish “Colonel” on J2 penetration of *H. schachtii* populations into roots of 7-day-old oilseed radish “Colonel”. Comparisons were carried out within the same population across different treatments indicated by lowercase letters or between different populations within the same treatment indicated by uppercase letters (Wilcoxon test, *t*-test, *p* < 0.05, *n* = 6).

The bioassay evaluating the infectivity of *H. schachtii* populations into the resistant oilseed radish “Colonel” demonstrated significant differences in penetration rates among the studied populations. In sterilized water, the Acholshausen population exhibited the highest average penetration rate, while the Kerpen-Buir population showed the lowest (Figure 2).

Rhizosphere microbial attachment to the J2 cuticle significantly reduced the number of penetrated J2s in the Acholshausen population compared to sterilized J2s (Figure 2).

For J2s treated with rhizosphere microbes, penetration levels were highest in the Ireland population and lowest in the Acholshausen population (Figure 2).

Notably, the microbial attachment did not influence root penetration by J2s from the Kerpen-Buir, Ireland, or Elsdorf populations (Figure 2).

### 3.3. The Penetration of H. schachtii Populations into Oilseed Radish Roots, with and Without Attachment of Rhizosphere Microbes from 69-Day-Old Plants

**Figure 3:**
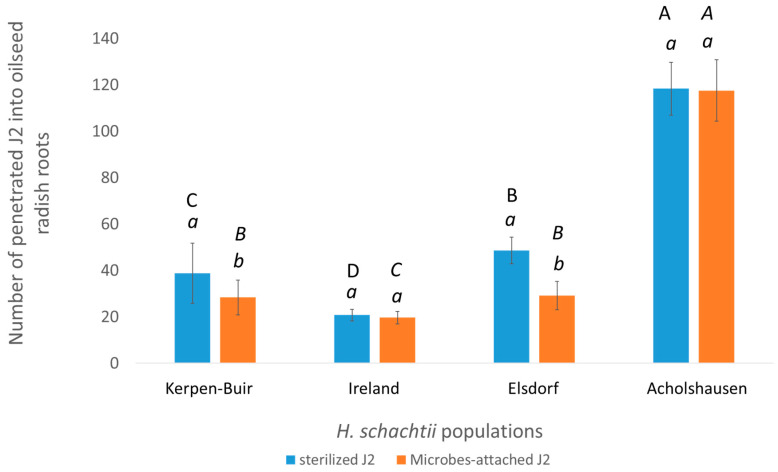
The effect of rhizosphere microbes isolated from 69-day-old resistant oilseed radish “Colonel” plants on J2s penetration of *H. schachtii* into the roots of 7-day-old oilseed radish “Colonel”. Comparisons were made within the same population across different treatments indicated by lowercase letters and between populations within the same treatment indicated by uppercase letters (Wilcoxon test, *t*-test, *p* < 0.05, *n* = 6).

Similar to the previous results, the four studied populations of *H. schachtii* differed in their penetration rates into the resistant oilseed radish “Colonel”. The penetration rate of sterilized J2s was highest in the Acholshausen population and lowest in the Ireland population (Figure 3).

The attachment of rhizosphere microbes to the J2 cuticle reduced the number of infected J2s in both the Elsdorf and Kerpen-Buir populations (Figure 3).

The number of treated J2s was highest in the Acholshausen population and lowest in the Ireland population. Although the penetration levels of sterilized nematodes showed a significant difference between the Elsdorf and Kerpen-Buir populations, the average number of infected treated J2s was similar between these two populations (Figure 3).

The microbial attachment did not affect root penetration by J2s from the Ireland and Acholshausen populations (Figure 3).

### 3.4. The Relative Abundance of Rhizosphere Bacteria Attached to J2s of H. schachtii Populations

**Figure 4:**
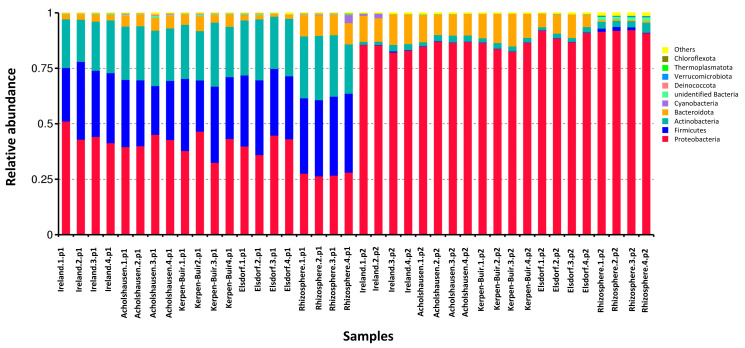
Relative abundances of rhizosphere bacteria at the phylum level, isolated from 39-day-old (p1) and 69-day-old (p2) resistant oilseed radish (Colonel) and attached to J2s originating from four populations of *H. schachtii*. Each population is represented by four sample replicates per treatment. The bar chart illustrates the top 11 bacterial communities with the highest relative abundances.

The relative abundances of bacterial phyla in rhizosphere microbes differed significantly between 39-day-old (p1) and 69-day-old (p2) plants (Figure 4).

In the rhizosphere of 69-day-old plants, Proteobacteria dominated, with relative abundances ranging from 86.45% to 91%. In contrast, the rhizosphere of 39-day-old plants exhibited a more diverse composition, with Proteobacteria, Firmicutes, and Actinobacteria accounting for 25.48–36.4%, 34.03–35.4%, and 20.02–29.12%, respectively.

This variation was mirrored in bacterial attachment to J2 cuticles of the four *H. schachtii* populations across the two treatments. For the Ireland population, bacteria from Proteobacteria, Firmicutes, and Actinobacteria attached to J2s treated with rhizosphere microbes from 39-day-old plants at rates of 40.95–50.05%, 29.575–35.03%, and 19.11–22.75%, respectively. With microbes from 69-day-old plants, Proteobacteria dominated at 84.1%, while Bacteroidota ranged from 11.3 to 13.65%.

Similar trends were observed in the other populations. For the Acholshausen population, J2s treated with rhizosphere microbes from 39-day-old plants had attachment rates of 38.22–44.59% for Proteobacteria, 22.75–32.76% for Firmicutes, and 22.75–25.025% for Actinobacteria. In the 69-day treatment, Proteobacteria increased to 93.27–95.55%, while Bacteroidota ranged from 9.1 to 10.92%.

In the Kerpen population, J2s treated with rhizosphere microbes from 39-day-old plants showed attachment rates of 31.85–45.5% for Proteobacteria, 22.75–34.125% for Firmicutes, and 22.75–29.575% for Actinobacteria. Under the 69-day treatment, Proteobacteria reached 91–95.55%, while Bacteroidota ranged from 9.1 to 16.38%.

Lastly, in the Elsdorf population, J2s treated with rhizosphere microbes from 39-day-old plants had Proteobacteria, Firmicutes, and Actinobacteria at proportions of 35.035–44.13%, 28.21–34.125%, and 27.3–36.4%, respectively. In the 69-day treatment, Proteobacteria dominated at 86.45–91%, while Bacteroidota ranged from 5.46 to 11.375%.

### 3.5. Alpha Diversity of Rhizosphere- and J2-Attached Bacterial Communities

**Figure 5:**
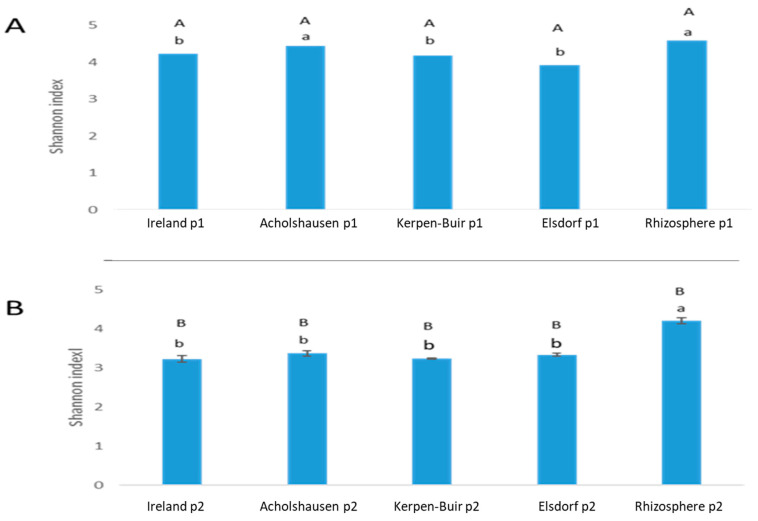
Shannon indices calculated for bacterial diversity of 16S rRNA, based on Illumina sequencing of rhizosphere bacteria isolated from 39-day-old (**A**) and 69-day-old (**B**) resistant oilseed radish “Colonel”. The bacteria were attached to J2s originating from four populations of *H. schachtii*. Each population is represented by four sample replicates per treatment. Different lowercase letters indicate significant differences in diversity among population-attached bacteria and rhizosphere bacteria within the same treatment, while different uppercase letters indicate significant differences between the two treatments for the same population or rhizosphere bacteria (Wilcoxon test, *t*-test, *p* < 0.05, *n* = 4).

The comparison of Shannon index values across treatments revealed that bacterial diversity in the rhizosphere and those attached to *H. schachtii* populations was higher in the first treatment compared to the second treatment (Figure 5).

In the first treatment, the highest Shannon index values were observed in the rhizosphere bacterial communities (4.687) and in the bacteria attached to J2s of the Acholshausen population (4.375). In contrast, lower values were recorded for the bacterial communities attached to J2s of the other three populations, ranging between 3.75 and 4.06 (Figure 5).

In the second treatment, alpha diversity was consistently higher in the rhizosphere bacteria than in the communities attached to the nematode cuticle. The Shannon index reached its maximum in the rhizosphere bacterial community (4.06), while the lowest values were observed in the bacterial communities attached to J2s of the four populations, ranging from 3.125 to 3.43 (Figure 5).

### 3.6. Beta Diversity of Rhizosphere- and J2-Attached Bacterial Communities

The UniFrac metric was employed to evaluate the phylogenetic distances of bacterial species within the analyzed communities. Weighted UniFrac values incorporate both species abundance and presence/absence, whereas unweighted UniFrac values focus solely on species presence or absence.

**Figure 6:**
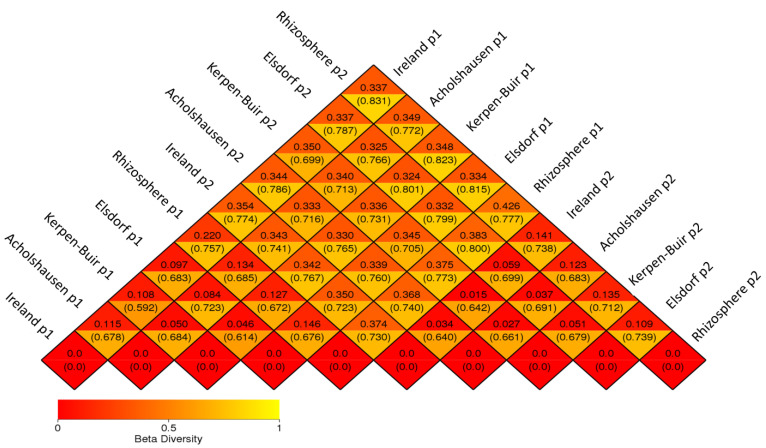
Heatmap of beta diversity for rhizosphere bacteria isolated from 39-day-old (Rhizosphere 1) and 69-day-old (Rhizosphere 2) resistant oilseed radish “Colonel” and attached to J2s originating from four populations of *H. schachtii*. Each population is represented by four sample replicates per treatment. The two values in each grid cell represent the weighted (quantitative) and unweighted (qualitative) UniFrac distances, respectively.

In the first treatment, weighted UniFrac values for rhizosphere–population comparisons ranged from 0.127 (Rhizosphere–Kerpen) to 0.220 (Rhizosphere–Ireland). Beta diversity of attached bacteria among populations showed weighted UniFrac distances ranging from 0.046 (Kerpen–Elsdorf) to 0.115 (Ireland–Acholshausen) (Figure 6).

For rhizosphere–population comparisons, unweighted UniFrac values ranged from 0.612 (Rhizosphere–Kerpen) to 0.757 (Rhizosphere–Ireland). Among populations, unweighted UniFrac values ranged from 0.614 (Kerpen–Elsdorf) to 0.723 (Acholshausen–Elsdorf) (Figure 6).

In the second treatment, weighted UniFrac values for rhizosphere–population comparisons ranged from 0.109 (Rhizosphere–Elsdorf) to 0.141 (Rhizosphere–Ireland). Among populations, weighted UniFrac values for the attached bacteria ranged from 0.015 (Kerpen–Ireland) to 0.059 (Ireland–Elsdorf).

Unweighted UniFrac values for rhizosphere–population comparisons in the second treatment ranged from 0.683 (Acholshausen–Rhizosphere) to 0.739 (Elsdorf-Rhizosphere). Among populations, unweighted UniFrac values ranged from 0.640 (Acholshausen–Ireland) to 0.723 (Ireland–Elsdorf) (Figure 6).

When comparing samples between the first and second treatments, the weighted UniFrac distance between Rhizosphere1 (39-day-old rhizosphere) and Rhizosphere2 (69-day-old rhizosphere) was 0.426, while the unweighted UniFrac value was 0.777.

For population comparisons across treatments, weighted UniFrac values ranged from 0.332 (Elsdorf1–Elsdorf2) to 0.354 (Ireland1–Ireland2), while unweighted UniFrac values ranged from 0.716 (Acholshausen1–Acholshausen2) to 0.799 (Elsdorf1–Elsdorf2) (Figure 6).

## 4. Discussion

The attached rhizosphere bacterial community to the second-stage juveniles (J2s) varied among the four studied populations of the beet cyst nematode *H. schachtii*. High diversity in the nematode cuticle among individuals is determined because of the variations in components such as collagen and glycans [32,33,34,35,36,37,38]. Such variability in the structural characteristics of nematode cuticles leads to differences in microbial attachment among nematode populations [32,39,40]. For instance, the bacterial attachments by *Pasturia penetrans* and *Rothia* sp. differed among populations of *Meloidogyne* and *Pratylenchus neglectus*, respectively [22,23].

Receptors on the nematode surface interact with specific components of microbial attachers, facilitating the attachment process [37,41]. Variations in the genetic structure of the genes responsible for encoding these receptors lead to differences in bacterial attachment within and among nematode populations [22]. One notable effector gene associated with microbial attachment to the nematode cuticle is the fatty acid- and retinol-binding protein (*far-1*). Silencing the *far-1* gene has been shown to reduce the attachment of *P. penetrans* to *Meloidogyne incognita*, highlighting its role in this interaction [42].

Consistent with this, variations in bacterial attachment have been reported among populations of *Pra. neglectus*, which differ in the structural composition of the *far-1* gene [22]. In further research, distinct gene expression profiles were observed in newly hatched J2s of *H. schachtii* across populations. Such variability in the genes encoding receptors that mediate microbial attachment may underlie the observed differences in rhizosphere bacterial associations among the four studied populations.

In addition to the variability in the bacterial attachment, the studied populations of *H. schachtii* differed significantly in the number of sterilized J2s that penetrated host roots.

PPNs respond to root exudate signals through host-specific gene expression patterns [43]. Genetic variation among nematode populations plays a crucial role in shaping plant–nematode interactions [24,25,28,44,45]. Previous studies have demonstrated population-specific gene expression in *Heterodera glycines* before infection and during the early stages of parasitism, highlighting the role of genetic variability in shaping host–nematode interactions [45].

Given the variability in gene expression observed among the J2s of the studied *H. schachtii* populations, differences in the rate of J2 penetration into host roots were anticipated. Notably, a variation in the structure of the parasitism gene *vap1* was identified among the four populations [25,46]. The *vap1* gene encodes variants with amino acid sequence differences in their exon regions, potentially leading to functional alterations. Such sequence variability likely stems from gene fragments exhibiting population-specific divergence within *H. schachtii* [24].

In the first experiment, the Acholshausen population showed the highest number of J2s penetrating plant roots. However, the attachment of rhizosphere microbes from 39-day-old plants significantly reduced J2 penetration, highlighting the crucial role of rhizosphere microbes from the resistant oilseed radish in suppressing J2s of *H. schachtii*.

The rhizosphere provides a critical niche for the most destructive PPNs, including cyst, root-knot, and root-lesion nematodes, as their life cycles commence and terminate within this zone [1]. The biological and chemical composition of the rhizosphere profoundly influences the degree of nematode suppression. When the rhizosphere is colonized by microbes that function as nematode parasites or predators, plant protection is enhanced, reducing nematode infections. However, the biological efficacy of these microbes is closely affected by the virulence of the nematode population, which, in turn, is primarily determined by its genetic structure [22,23].

The attachment of rhizosphere microbes from 39-day-old plants to J2s of the other three populations did not significantly reduce the number of penetrated individuals. This finding underscores the population-specific nature of microbial attachment and its influence on nematode penetration. These results suggest that the interaction between rhizosphere microbes and nematodes is influenced by the genetic and physiological characteristics of the nematode populations, which dictate their susceptibility to microbial suppression.

The attachment of rhizosphere microbes from 69-day-old plants to J2s reduced the number of penetrated individuals from the Elsdorf and Kerpen populations, highlighting the role of plant age in shaping the effects of rhizosphere microbes.

The microbial analysis revealed significant differences in the bacterial community composition between the rhizospheres of 39-day-old and 69-day-old oilseed radish plants. While bacterial species from the phyla Proteobacteria, Firmicutes, and Actinobacteria were dominant in the rhizosphere of 39-day-old plants, Proteobacteria dominated the rhizosphere of 69-day-old plants. This shift in microbial composition can be attributed to age-related changes in plant secretions, which influence the structure and functionality of the rhizosphere microbial community [46]. Previous research has demonstrated that the microbial communities associated with older plants tend to be more specialized than younger plants [47]. These differences in bacterial community composition contributed to variations in the bacterial attachment to the J2s cuticles of different nematode populations, subsequently altering their nematode-suppressive effects. Notably, while the rhizosphere microbes of 39-day-old plants had no significant impact on the J2 penetration of the Elsdorf and Kerpen populations, those of 69-day-old plants effectively suppressed their penetration. Conversely, the rhizosphere microbes of 69-day-old plants had no suppressive effect on the Acholshausen population, underscoring the complex interplay between the plant age, microbial community composition, and nematode population-specific responses.

The variation in the number of sterilized penetrated J2s was not in the same line across the two treatments. In the first experiment, the Kerpen population had the lowest number of penetrated J2s, while in the second experiment, Ireland had the lowest one. Interestingly, the number of penetrated J2s varied within the same population between the two treatments, except for the Acholshausen population, which consistently showed the highest number of penetrated J2s across both experiments.

These findings suggest that the differences in J2s suppression between treatments cannot solely be attributed to variations in the bacterial community. Instead, they may also reflect genetic differences among individuals within the same population. Such genetic variability could influence the nematode–microbe interactions within a population, leading to population-specific differences in plant-nematode interactions.

In the second treatment, the number of sterilized penetrated J2s varied between the Elsdorf and Kerpen populations. However, this difference was masked by microbial attachment to their J2s cuticles. This observation underscores the critical role of rhizosphere microbes in influencing plant–nematode interactions, either directly by suppressing the nematode activity or enhancing plant defense mechanisms [16].

The alpha diversity, as measured by the Shannon Index, differed significantly between the rhizosphere microbiomes of the 39-day-old and 69-day-old plants, with higher diversity observed in the rhizosphere of the younger plants. This difference is attributable to the variation in root secretions influenced by plant age, which impacts the composition and diversity of the microbial community [46].

In the first treatment, the Shannon Index of the bacterial communities attached to J2s varied between the rhizosphere and the J2-attached bacteria, except for the Acholshausen population. This result aligns with findings from previous research, which highlighted the specificity of microbial attachment [22]. In that study, nematodes from the *Pra. neglectus* population were incubated separately in two bacterial solutions (*Rothia* sp. and *E. coli*). Compared to *E. coli*, *Rothia* showed a higher attachment rate to the nematode cuticle, indicating that microbial attachment depends on the bacterial species and receptor structures on the nematode cuticle. Unlike the other three populations, the bacterial community attached to the Acholshausen J2s exhibited the highest Shannon index and closely resembled the rhizosphere community. This result aligns with the result that showed the highest number of CFUs formed by bacteria attached to J2s from the Acholshausen population. It suggests that a higher receptor diversity on the J2 cuticle is likely related to the greater diversity of attached bacteria. 

The Shannon Index in the second treatment revealed significant differences between the bacterial communities in the rhizosphere and those attached to J2s across the four populations. The low bacterial diversity in the rhizosphere may drive a higher level of specialization in the microbes attached to the nematode, even in the Acholshausen population, which is expected to exhibit a higher receptor diversity.

The microbial influence on nematode penetration in the first treatment was similar across the Irland, Elsdorf, and Kerpen populations, where the Shannon Index of the attached bacteria did not vary significantly. However, despite the Shannon Index of the attached bacteria being similar across these three populations in the second treatment, their effect on nematode penetration diverged. Suggesting that the impact of microbial attachment on nematode penetration is not solely determined by the bacterial species but also by the defensive capacity of the juvenile nematodes to the attached microbes. This complexity highlights the role of nematode resistance mechanisms in mediating the effectiveness of microbial interactions in suppressing nematode penetration.

The alpha diversity of the rhizosphere bacterial community was higher in 39-day-old oilseed radish plants compared to 69-day-old plants. This change in bacterial diversity is related to age-related differences in root exudate composition, which influences the microbial community structure [46]. As a result, differences in the Shannon Index of the bacteria attached to J2s within the same population were observed between the first and second treatments, Considering the differences in the physiological and genetic structures among J2s within the same population. This genetic variability can influence their susceptibility to rhizosphere bacteria and their ability to penetrate plant roots, highlighting the importance of understanding these intra-population differences for more precise management of nematode populations.

Illumina sequencing analysis incorporated beta diversity to evaluate pairwise comparisons of bacterial communities across treatments. In both treatments, distinct variations in dominant bacterial abundance were observed in rhizosphere–population comparisons, supporting the specificity of rhizosphere bacterial attachment to the J2 cuticle. In addition, the elevated unweighted UniFrac values in population–population comparisons highlighted differences in the bacterial species attached to nematode populations.

Plant age influences the abundance and composition of rhizosphere bacteria, resulting in differences in weighted and unweighted UniFrac values across rhizosphere and population comparisons between the two treatments. This highlights the significant impact of the plant developmental stage on microbial community composition and its interaction with nematode populations.

In the current study, the fungal community was not analyzed due to the insufficient quantity of fungal isolates in the rhizosphere of 39-day-old plants, which did not meet the detection threshold for Illumina sequencing. Consequently, the study focused solely on the effects of rhizosphere bacteria on nematode penetration.

Older plants exhibit resistance to plant-parasitic nematodes because of the enhanced strength of their defense mechanisms. However, the observed changes in plant resistance to nematodes may also be influenced by the quantity and quality of rhizosphere microbial isolates. Rhizosphere-colonizing microbes can antagonize nematodes directly or function as endophytes, enhancing the plant’s defense against PPNs.

In conclusion, the rhizosphere microbes associated with resistant catch crops are essential in controlling the beet cyst nematode *H. schachtii*. However, the effectiveness of these microbial rhizosphere-colonizers is affected by plant age and the aggressiveness of the nematode population, which are critical factors in determining their efficacy against this pest.

## Data Availability

The original contributions presented in this study are included in the article. Further inquiries can be directed to the corresponding authors.
